# Epidemic Cholera

**Published:** 1848-10

**Authors:** 


*Epidemic Cholera.*—We quote from our much esteemed contemporary, the Annalist, the following summary of the progress of Epidemic Cholera in Europe.—

"The alarming fact is stated, that in St. Petersburg, up to the last accounts, 17,000 cases of cholera had occurred, of which, 10,138 had proved fatal. The deaths were 57 in every 100; the cures only 26 in every 100. At Moscow there had been 9751 cases, and 4,309 deaths. In various parts of Bessarabia the disease was advancing rapidly; and in Fontarabia, Bucharest, Jassy, and other places, the greatest alarm prevailed. At Jassy the deaths were at 100 to 130 per day. At Cairo it had appeared in a form of peculiar malignity. Its ravages had been confined to the city itself; and although the medical men had treated the disorder with every possible care, not one case had been saved, but every patient had died in the course of a few hours after the first attack. In St. Petersburgh the disorder is now abating. It has, however, made its appearance in Berlin, where, at the last accounts, there had been four cases, all fatal. It has also broken out mildly at Vienna.

